# Fra-1 governs cell migration via modulation of CD44 expression in human mesotheliomas

**DOI:** 10.1186/1476-4598-6-81

**Published:** 2007-12-21

**Authors:** Maria E Ramos-Nino, Steven R Blumen, Harvey Pass, Brooke T Mossman

**Affiliations:** 1 Department of Pathology, University of Vermont College of Medicine, Burlington VT 05405, USA; 2 Department of Cardiothoracic Surgery, New York University School of Medicine, New York, NY 10016, USA

## Abstract

Silencing of Fra-1, a component of the dimeric transcription factor, activator protein-1 (AP-1), inhibits mRNA expression of c-*met *and *cd44 *in rat mesothelioma cells and is causally linked to maintenance of the transformed phenotype. However, the mechanisms of Fra-1 regulation and Fra-1 regulated gene expression in human malignant mesothelioma (MM) are unclear. We first show in a panel of human MM cells that Fra-1 mRNA expression in MM is complex and regulated by extracellular signal-regulated kinase (ERK1, ERK2), Src, and phosphatidyl-inositol-3-kinase (PI3K) pathways in a tumor-specific fashion. Cell lines with PI3K-dependent Fra-1 expression were SV40 positive and expressed the lowest basal Fra-1 levels. Levels of Fra-1 expression correlated with amounts of CD44 expression that were greater in simian virus 40 negative (SV40-) MM cells. Using dominant negative (dn), short hairpin (sh) and small interference (si) RNA constructs, we next demonstrate that expression of CD44, the principal hyaluronic receptor in MMs, correlates with Fra-expression in both simian virus 40 positive (SV40+) and SV40- MMs. Moreover, both Fra-1 and CD44 expression are linked to cell migration in SV40- MM cells. Lastly, in contrast to normal lung tissue, tissue microarrays revealed that Fra-1 was expressed in 33 of 34 human MMs, and that all CD44+ tumors were SV40-. These results suggest that Fra-1 is associated with cell migration in human MMs and that Fra-1 modulation of CD44 may govern migration of selected MMs.

## Background

Malignant mesothelioma (MM) is an insidious tumor associated historically with occupational exposure to asbestos [1,2]. Recently, infection by simian virus 40 (SV40) has been implicated as a contributory factor in the development of MMs [3,4] but these findings are controversial [5-7]. The average survival of patients is less than 1 year after initial diagnosis of MM, and no successful treatment options exist for the majority of patients [1,3]. These pleomorphic tumors are unique in that they have a long latency period (average of 30+ years) and various pathologies (epithelial, sarcomatous and mixed) that complicate their diagnosis and may govern their prognosis [1,3].

Although the mechanisms of development of MM are obscure, the initiation of signaling events after interaction of mesothelial cells with asbestos fibers or infection by SV40 may result in transactivation of genes governing cell proliferation and other properties of neoplastic cells [2,8,9]. The transcription factor, activator protein-1 (AP-1) consists of members of the Jun (c-Jun, JunD, JunB) and Fos (c-Fos, FosB, Fra-1, Fra-2) family of early response protooncogenes [10,11] and is a major target of asbestos-induced cell signaling via activation of mitogen activated protein kinases (MAPK) [12,13].

In comparison to other Jun and Fos family members, increases in Fra-1 expression by asbestos are protracted in rodent lung epithelial [14] and pleural mesothelial cells and are critical in maintenance of the malignant phenotype of rat MMs [15]. Moreover, *cd44*, which encodes the principal hyaluronic acid receptor in a variety of cell types, is a *fra-1 *regulated gene in rat MMs [16].

CD44 is a type I transmembrane glycoprotein (85–200 kDa) and functions as the major cellular adhesion molecule for hyaluronic acid (HA), a component of the extracellular matrix (ECM). CD44 is expressed in most human cell types and is implicated in a wide variety of physiological and pathological processes, including lymphocyte homing and activation, wound healing, cell migration, tumor cell growth, metastasis [17,18] and chemoresistance [19]. The CD44 gene consists of at least 19 exons, of which 12 can be alternatively spliced [18], and this differential gene expression through alternative splicing is important to various physiological and pathological conditions [20]. The most common isoform expressed in a variety of cell types is CD44s (standard). The distribution of the CD44 variants is usually restricted, and some variants are only expressed in certain tumor cells where their expression can confer metastatic properties [21].

The CD44 hyaluronic acid receptor is upregulated in human MMs [22], and increased hyaluronic acid in pleural fluid and serum is used both as a diagnostic and prognostic indicator of MM [23-27]. In a previous study, it was found that MM cell lines that expressed the highest amount of CD44 receptor showed increased proliferation and haptotactic migration when stimulated with low molecular weight hyaluronic acid [28]. Furthermore, the use of a monoclonal antibody against CD44 inhibited proliferation by 12–40% and migration by 10–35% in the MM cell lines that were studied [28]. The goal of studies here was to elucidate cell signaling pathways leading to transactivation of CD44 by Fra-1 and their functional ramifications on migration of both SV40+ and SV40- human MM cells. We first established that Fra-1 expression is inducible by serum and is heterogeneous in different MM cells when modulated by inhibitors of the P13K, Src or ERK1/2 pathways. Levels of Fra-1 correlated with CD44 protein levels that were higher in SV40- MMs. The functional significance of *Fra-*1*-*dependent CD44 expression was determined in high CD44-expressing SV40- MM cells using small hairpin (sh) RNA interference constructs. These experiments showed that inhibition of *Fra-1 *or *CD44 *significantly curtailed MM cell migration. More importantly, Fra-1 overexpression was observed in 33 of 34 human MMs in tissue arrays and all CD44+ tumors were SV40-.

## Results

### Inhibition of PI3K, Src or the ERK1/2 pathway diminishes *Fra-1 *expression, transactivation and protein levels in human MM cells in a tumor-specific manner

**Figure 1:**
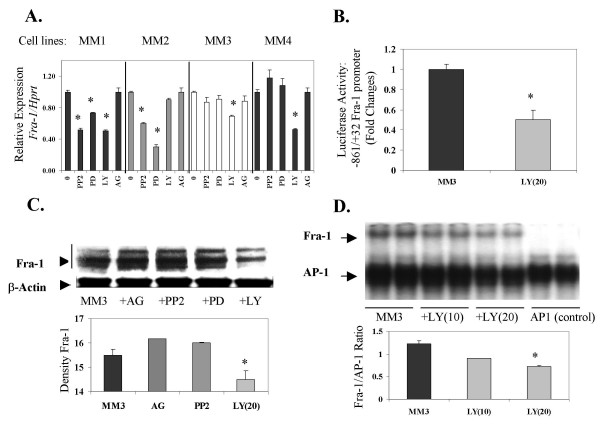
ERK1/2, Src and PI3K regulation of Fra-1 expression in MM cell lines is tumor line-specific. A. RT-QPCR showing Src, ERK1/2, and PI3K-dependent decreases in *Fra*-1 in MM1, Src and ERK1/2-dependent decreases of *Fra-*1 in MM2, and PI3K-dependent decreases in *Fra-*1 in lines MM1, MM3 and MM4. B. Luciferase assays show PI3K-dependent *Fra*-1 promoter activation in MM3 cells. C. Western blot analysis of Fra-1 shows inhibition by the PI3K inhibitor, LY294002 at 20 μM. D. EMSA shows a dose dependent decrease in Fra-1 in the AP-1 complex after treatment of the MM3 line with the PI3K inhibitor, LY294002 at 10 and 20 μM * = P ≤ 0.05 in comparison to untreated control (0).

We first focused on whether heterogeneous pathways of Fra-1 regulation occurred in human MM cells using inhibition of upstream signaling cascades. In Figure 1A, we observed that the PI3K inhibitor, LY294002 (LY 20 μM), caused significant reduction of *Fra-1 *mRNA levels in 3 of the 4 MM lines initially examined, whereas addition of AG1478 (AG 10 μM), an inhibitor of EGFR phosphorylation, had no effects on *Fra-1 *expression. Two of the 4 MM lines showed inhibition of *Fra-1 *expression after pretreatment with PP2 (10 μM) or PD98059 (PD, 30 μM), respectively, suggesting additional pathways of *Fra-1 *modulation in some MMs. In MM3 cells in which *Fra-1 *mRNA levels were diminished significantly only after inhibition of the PI3K pathway, transactivation of *Fra-1 *dependent gene expression (Figure 1B) and protein levels (Figure 1C) were also inhibited selectively by LY294002 (20 μM). In accordance with data presented in Figure 1A, pre-addition of the EGFR kinase inhibitor, AG1478 (10 μM) did not affect Fra-1 protein levels (Figure 1C). In Figure 1D, we also show using an EMSA super-shift assay that inhibition of the PI3K pathway by LY294002 (10 and 20 μM) causes reduced expression of Fra-1 in the AP-1 complexes of these cells in a dose-related fashion.

### Levels of Fra-1 and CD44 expression are greater in SV40- human MM cells

**Figure 2:**
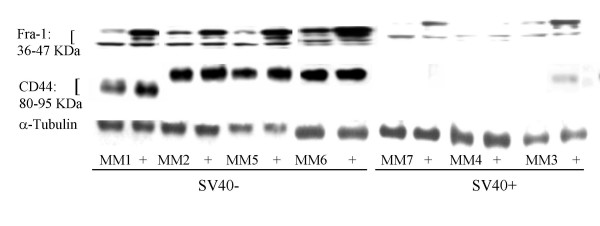
MMs with high basal Fra-1 expression have also higher CD44 expression. Western blots show constitutive levels of Fra-1 (three bands show different levels of phosphorylation), CD44, and levels of α-Tubulin (control for protein loading) in confluent cells maintained for 24 h in serumless medium or after 4 h with the addition of 10% FBS (columns +).

Western blot analyses were performed on a panel of 7 MM cell lines at near confluency to determine possible correlations between expression of these proteins in control cells and with addition of serum (+ lanes) for 4 h (Figure 2). Fra-1 was inducible in all MMs after addition of serum. In the 3 SV40 + cell lines, both Fra-1 and CD44 protein were markedly reduced in comparison to SV40- cell lines in the presence or absence of serum.

### Levels of Fra-1 and modulation of its upstream regulators are associated with CD44 expression levels

**Figure 3:**
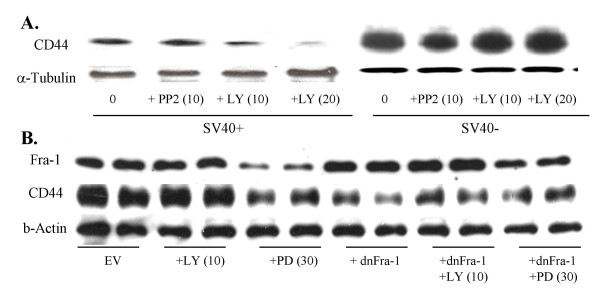
CD44 expression is dependent on Fra-1 and the pathways that control Fra-1 expression. (A) Western blot showing the effect of the PI3K and Src inhibitors in the CD44 expression of an SV40+ and SV40- cell line. (B) Western blots showing that CD44 expression in the SV40- MM line follows the pattern of its Fra-1 expression. The use of a dominant negative construct for Fra-1 (dnFra-1) decreases CD44 expression, but not further decreases are observed after PD98059 or LY294002 is added to the dnFra-1 stable cell line.

Here we examined levels of endogenous CD44 in SV40+ and SV40- MM lines after addition of LY294002 (Figure 3). These studies revealed that the PI3K inhibitor LY294002 diminished CD44 protein in a dose-related fashion in the SV40+ line, but not in the SV40- MM lines. The Src inhibitor, PP2, did not affect CD44 expression in the SV40+ MM line, but decreased CD44 expression in the SV40- MM line (Figure 3A). CD44 protein was decreased after addition of the PD98059 MEK1 inhibitor and in a SV40- cell line transformed with a dn-Fra-1 construct (Figure 3B). However, CD44 depletion was not increased synergistically after PD98059 or LY294002 was added to the dnFra-1 stable cell line (Figure 3B). These results show the complexity of Fra-1 regulation in MM and suggest that Fra-1, Src, ERK1/2-and PI3K are also modulators of CD44 expression in different MM lines. CD44 promoter analysis does not suggest the direct participation of Fra-1 in CD44 expression (data not shown).

**Figure 4:**
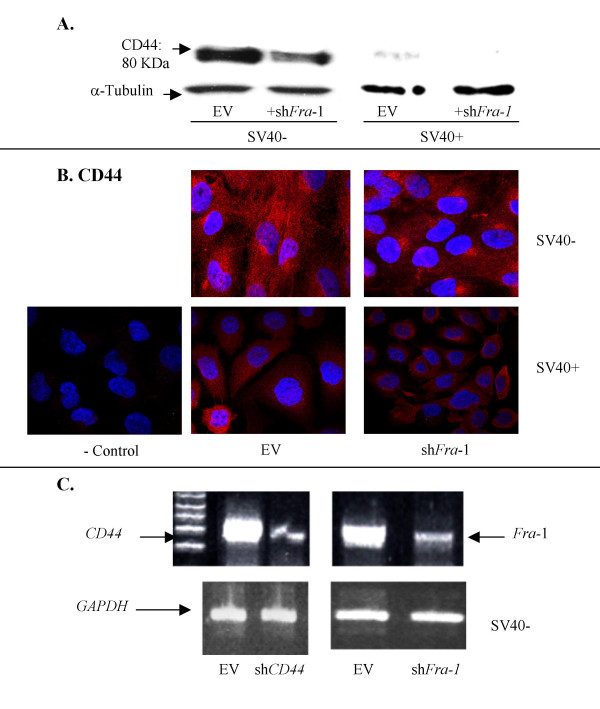
Use of shRNA constructs to knock-down *Fra*-1 confirm *Fra*-1-dependent CD44 expression in MMs. **A**. Western blots of SV40- (MM1) and SV40+ (MM3) lines showing empty vector (EV) controls or RNAi sh*Fra*-1 constructs. **B**. Immunofluorescence image showing CD44 (red) levels MM cell lines. **C**. Agarose gels on an SV40- cell line showing the efficiency of the RNAi constructs (sh*Fra*-1 and sh*CD44*) on knockdown of *CD44 *and *Fra-*1 measured by a semi-quantitative RT-PCR reaction and using *GAPDH *as a control.

To determine a role of Fra-1 in CD44 expression, we next developed a sh*Fra*-1 construct and verified in both an SV40+ and SV40- MM line that CD44 expression was reduced (Figure 4A). We further showed that sh*Fra*-1 in the SV40- line reduced CD44 expression (red) using immunochemistry and CSLM, whereas the SV40+ line had low basal levels of CD44 (Figure 4B). The efficiency of the sh*Fra*-1 construct and a *shCD44 *construct for inhibition of CD44 expression in functional assays below is depicted in Figure 4C using semi-quantitative PCR in a SV40- MM line.

### sh*Fra-1 *and shCD44 constructs inhibit motility of MM cells

**Figure 5:**
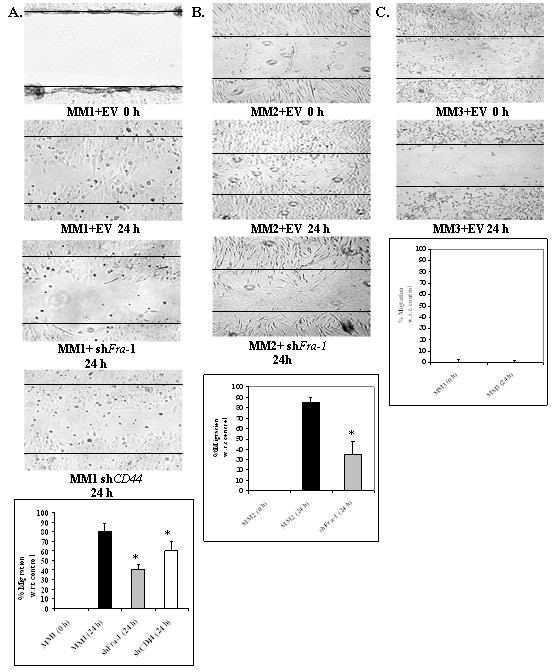
Fra-1 and CD44 are critical to MM cell migration. Wound assays showed that in the SV40- cell lines MM1 (panel A) or MM6 cell line (panel B) sh*Fra*-1 and sh*CD44 *transfected cells showed less migration than empty vector (EV) controls. * = P ≤ 0.05 in comparison to 24 h controls.

Two Fra-1 and CD44 expressing SV40- MM lines (MM1, MM2) and one SV40+ MM cell line (MM3) were used to demonstrate that Fra-1 and CD44 were causally linked to cell migration. In Figure 5A, migration was compared in stable MM1 lines transfected with sh*Fra-1*, sh*CD44*, or the empty vector (EV) control. These studies showed that after addition of serum for 24 h, numbers of migrating cells in the sh*Fra-1 *or sh*CD44 *cell lines were significantly decreased (p < .05) in comparison to the MM1 (EV) cells. These results were confirmed using sh*Fra-1 *transformed MM2 and MM2 (EV) stable cell lines (Figure 5B). In Figure 5C, no migration was detected in stable MM3 lines transfected with sh*Fra-*1 or the empty vector (EV).

### sh*Fra-1 *constructs reduce hyaluronic acid uptake of MM cells

**Figure 6:**
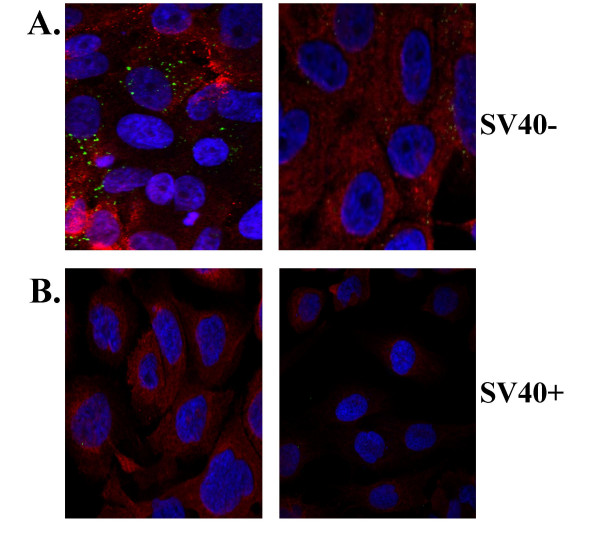
Immunofluorescence image shows greater uptake of BODIPY FL hyaluronic acid by MMs with higher Fra-1 expression levels (SV40-) (A). Internalization of the BODIPY FL HA probe (green) was visualized by fluorescence confocal microscopy. This internalization is reduced in the SV40+ line with low basal Fra-1 (red) expression (B) or those with high basal Fra-1 expression after Fra-1 knock-down (right panel).

CD44 is known to bind hyaluronic acid (HA), and is also thought to contribute to HA internalization. The binding of HA to CD44 stimulates cytoskeleton-mediated tumor cell migration [29]. In Figure 6 we show that the uptake of BODIPY FL hyaluronic acid (green) is decreased in SV40- MM cells transfected with sh*Fra-1 *(Fra-1 expression is shown in red) compared to MM cells transfected with empty vector (EV). Moreover, BODIPY FL hyaluronic acid did not get internalized in the SV40+ MM line.

### Tissue arrays demonstrate that Fra-1 is overexpressed in the majority of MMs

**Figure 7:**
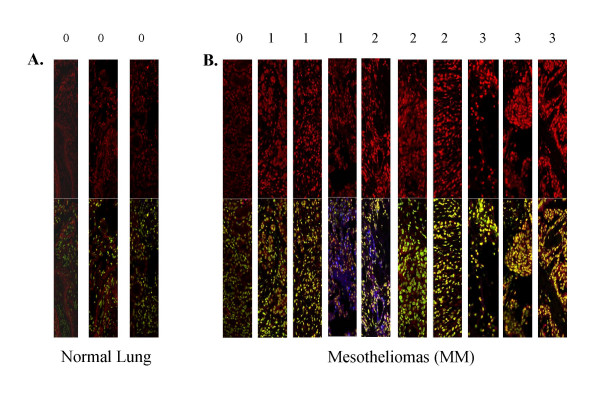
Tissue microarray slide showing that Fra-1 staining (intensity score of 1–3 compared to 0 as observed in 3 normal lungs) is expressed in the majority of human mesotheliomas (MM), (42/43). Images in A and B show immunostaining of Fra-1 (red) in the upper panel. Bottom panels show merged images of Fra-1, p-ERK1/2 and nuclear staining (SYTOX green).

We have shown previously that endogenous levels of Fra-1 are increased in human and rat MM cell lines as compared to normal rat mesothelial cells [15]. To confirm this in human MMs, tissue arrays containing normal lung (Figure 7A), and MMs (Figure 7B) were examined by multi-fluorescence approaches using antibodies for Fra-1 (red), phosphorylated (p)-ERK1/2 (blue) and SYTOXgreen (green) to detect nuclei. The images in Figure 7A and 7B are split into an upper portion showing Fra-1 expression, and a lower portion showing triple fluorescence studies for detection of p-ERK1/2 (blue), Fra-1 (red) and SYTOXgreen (green). In comparison to normal lungs (negative control), 33 out of 34 MMs stained positively for Fra-1 with different intensities. On a scale of 0–3 (0 = no expression, 1 = low expression, 2 = medium expression and 3 = high expression) using a blind coding system, ~3% MMs showed no Fra-1 immunoreactivity (score of 0), 29% were scored 1, 27% were scored 2 and 41% were scored 3. Fra-1 immunolocalization in MMs was either nuclear (41%), mainly nuclear with some cytoplasmic staining (32%), entirely cytoplasmic (6%), mainly cytoplasmic with some nuclear staining (18%) or negative (~3%). As shown in Figure 7 (lower panel), increased p-ERK1/2 immunoreactivity was observed in approximately 30% of tumors, and its localization was mainly cytoplasmic.

### Tissue arrays demonstrate that SV40 T-antigen expression in MM cells correlates with CD44- status

**Figure 8:**
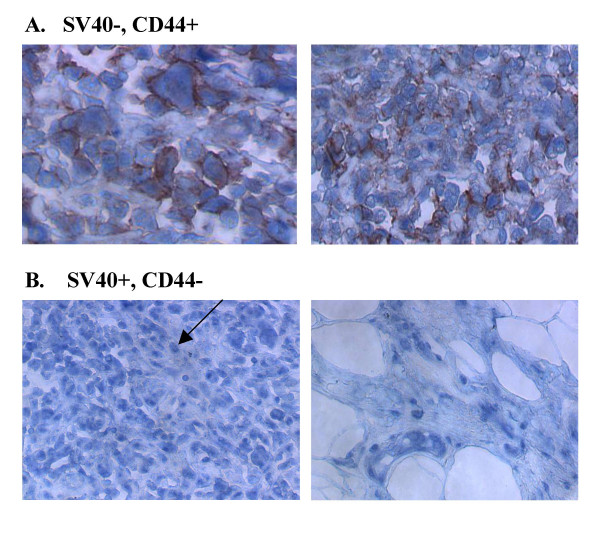
Examples of human MM in tissue microarrays co-stained using antibodies for SV40 T-antigen (blue/nuclear) and CD44 (red). Of the 34 MMs in the array, approximately 50% were SV40+, and of these SV40+ tumors (~100%) were CD44-. Of the 17 SV40- tumors, approximately 53% were CD44+ (all CD44+ tumors in the tissue array). **A**. Sample panel of SV40-, CD44+ tumors. **B**. Sample panel of SV40+, CD44- tumors. The arrow indicates nuclear SV40 T-antigen staining.

Using a similar tissue microarray set as used for Fra-1 expression above, we performed dual fluorescence studies on MMs using antibodies for SV40 T-antigen (blue/nuclear) and CD44 (red) (Figure 8). Of the 34 MMs, 17 (50%) were SV40+ and 8 (24%) were CD44+. Moreover, all SV40+ tumors were CD44-. Of the 17 SV40- tumors (50%), 9 (26%) were CD44-.

A side by side comparison of the Fra-1 array with the SV40/CD44 array showed that Fra-1 localization was primarily nuclear for all tumors with some cytoplasmic localization in CD44- tumors (Table 1).

**Table 1:** Cell localization of Fra-1 compared to SV40 and CD44 status in human MMs.

**SV40/CD44 Status**	**Fra-1 localization**			
	
	Nuclear (*)	**Cytoplasmic**	**None**	**Total**
-/-	5(56)	3(33)	1(11)	**9**
-/+	8(100)	0	0	**8**
+/-	12(71)	5(29)	0	**17**
+/+	0	0	0	**0**
**Total**	**1**	**10**	**9**	**34**

(*) = % of samples in the specific SV40/CD44 category.

## Discussion

The AP-1 family member, Fra-1, is up-regulated in several tumors, including stomach [30], esophageal [31], squamous cell carcinomas [32], thyroid [33,34], and breast tumors [32]. Although Fra-1 plays an important role in cell transformation and is upregulated by cigarette smoke, mitogens and phorbol ester tumor promoters [33-43], little is known about how this important protein is regulated in human tumors. Moreover, the functional ramifications of its expression and its mechanisms of action on individual tumor types are unclear.

Fra-1 expression and its activation by the ERK1/2 pathway have been well documented in several systems including rat MM cells [15]. More recently, gene profiling studies revealed that Fra-1 was an AKT-inducible gene in prostate cancer cells and vascular smooth muscle cells [36,39] and was a PI3K-dependent gene in human bronchial epithelial cells [43] and MM cells [38]. Here we present evidence that Fra-1 mRNA expression is complex in human MMs, involving activation of the ERK1/2, PI3K, and Src pathways in a tumor cell-specific manner. In contrast, levels of Fra-1 in MMs were not modified by pre-addition of an inhibitor of EGFR phosphorylation (AG1478) although it is known that EGFR phosphorylation leads to ERK1/2 activation in rodent mesothelial [13] and alveolar epithelial cells [44]. Moreover, both matrix metalloproteinase/EGFR/MAPK [45] and PI3K regulated AKT independent signaling pathways regulate Fra-1 induction by cigarette smoke [43].

We previously reported that increases in Fra-1 expression in rat mesotheliomas were causally linked to genes governing cell motility and invasion (*cd44 *and *met*) [16]. Other investigators also have reported that AP-1 mediated invasion of transformed fibroblasts requires increased expression of CD44 [46]. We hypothesize that the increased expression of Fra-1 in the AP-1 complex causes the activation of target genes that influence malignancy and invasion of human MMs. Here we present evidence that Fra-1 activation is through multiple survival pathways and that its expression governs human mesothelioma cell migration partially via indirect modulation of CD44 expression.

In human MM cell lines and tumors, the degree of constitutive Fra-1 expression is tumor line-dependent. The localization of Fra-1 in all tumors was primarily nuclear, but some cytoplasmic localization could be observed in CD44- tumors. Although the nuclear localization of any transcription factor is of critical importance for its function in downstream gene regulation, the cytoplasmic localization of Fra-1 could also have some impact in its function. Simultaneous nuclear and cytoplasmic localization of Fra-1 has been reported in breast malignancies [47], although its functional significance was unexplored.

We show here that low basal Fra-1 and CD44 levels correlated with SV40 positivity in MM cell lines and could be upregulated upon stimulation with serum. Previous studies have demonstrated that SV40 large T antigen can activate the PI3K pathway in different cell types [48,49], and, in line with these observations, we have shown that SV40+ MM cells have higher AKT activity [50,51]. Moreover, SV40+ MMs were more susceptible to killing when the PI3K pathway was inhibited. These studies suggest that SV40 positivity of MMs may render a survival advantage. However, CD44 does not contribute to the migration of SV40+ MM cells which may depend upon multiple mechanisms including voltage-gated sodium channels [52], expression of the EphA2 receptor [53], MAPK-regulated MMPs [54-56], and integrin or ECM composition and synthesis [57].

The presence and role of SV40 in human tumors is still very controversial, but SV40 T-antigen or DNA has been found in human MMs [5,58]. Results here suggest that SV40 is a contributor to the heterogeneity of migration responses found in human MMs.

The role of other Fra-1-dependent genes and signaling pathways should be explored in prevention and therapy of MMs of both SV40+ and SV40- MMs.

## Methods

### Human mesothelioma (MM) cell lines

Human pleural mesothelioma cell lines were isolated from patients at autopsy (from Dr. Michele Carbone, University of Hawaii, Honolulu, HI (MM3, MM4, MM7), and Dr. Luciano Mutti (Maugeri Foundation, Pavia, Italy) (MM1, MM6), or from surgical debulking of MMs from Dr. Harvey Pass (New York University, NY, NY) (MM2). The cell line MM5 (#CRL-2081) was obtained from the ATCC (Manassas, Virginia). All MM cell lines were tested for the mRNA expression of large and small T/t-antigen by PCR before each experiment. The MM1, MM2, MM5, and MM6 lines were negative for SV40 large T-antigen (SV40-), whereas MM3, MM4 and MM7 were SV40+. Cells were maintained in frozen stocks and propagated in DMEM/F12 medium (GIBCO BRL, NY) containing 10% fetal bovine serum (FBS), hydrocortisone (100 ng/ml), insulin (2.5 μg/ml), transferrin (2.5 μg/ml), and selenium (2.5 ng/ml) (Sigma, St Louis, MO).

### Small molecule inhibitors and chemicals

Stock solutions of all inhibitors were diluted in dimethyl sulfoxide (DMSO) and used at effective nontoxic concentrations as reported previously: The MEK1/2 inhibitor, PD98059, at 30 μM [15]; the EGFR inhibitor, AG1478, at 10 μM [13]; the PI3K inhibitor, LY294002, at 10 and 20 μM [59]; and the Src inhibitor, PP2, at 10 μM [44]. All chemicals were obtained from Calbiochem (La Jolla, CA). Control groups of cells also received DMSO (0.1%) in medium.

### Western blots analyses

Nearly confluent MM cells were washed 3 × with cold phosphate-buffered saline (PBS), scraped from culture plates, and collected by centrifugation at 14,000 rpm for 1 min. The pellet was resuspended in lysis buffer [20 mM Tris (pH 7.4), 1% Triton X-100, 10% glycerol, 137 mM NaCl, 2 mM EDTA, 25 mM β-glycerophosphate, 1 mM Na_3_VO_4_, 2 mM pyrophosphate, 1 mM PMSF, 10 μg/ml leupeptin, 1 mM DTT, 10 mM NaF, 1% aprotinin], incubated at 4°C for 15 min, and centrifuged at 14,000 rpm for 20 min. Protein concentrations were determined using a Bio-Rad assay (Bio-Rad, Hercules, CA). Twenty μg of protein in sample buffer [62.5 mM Tris-HCl (pH 6.8), 2% sodium dodecyl sulfate (SDS), 10% glycerol, 50 mM dithiothreitol, 0.1% w/v bromophenol blue] was resolved by electrophoresis in 10% SDS-polyacrylamide gels, and transferred to nitrocellulose using a semi-dry transfer apparatus (Ellard Instrumentation, Ltd., Seattle, WA). Blots were incubated in blocking buffer [Tris-buffered saline (TBS) containing 5% nonfat dry milk plus 0.1% Tween-20 (Sigma)] for 1 h, washed 3 × for 5 min each in TBS/0.1% Tween-20, and incubated at 4°C overnight with antibodies specific to CD44 and Fra-1 at a dilution of 1:500 (Santa Cruz Biotechnology Inc., Santa Cruz, CA). Blots were then washed 3 × with TBS/0.1% Tween-20 and incubated with a specific peroxidase-conjugated secondary antibody at a dilution of 1:5,000 (Amersham Pharmacia Biotech, Piscataway, NJ) for 1 h. After washing blots 3 × in TBS/0.1% Tween-20, protein bands were visualized with the LumiGlo enhanced chemiluminescence detection system (Kirkgaard and Perry Laboratories, Gaithersburg, MD) and quantitated by densitometry [44]. Blots were reprobed with an antibody to α-Tubulin in a dilution 1:1,000 (Santa Cruz Biotechnology, Santa Cruz, CA) or β-Actin at a dilution of 1:5,000 (Abcam Inc, Cambridge, MA) to validate equal loading between lanes [15].

### Luciferase Assay

Cells were transiently co-transfected with 2 μg reporter plasmid, human *Fra-1 *promoter-luciferase (-861/+32) (kindly obtained from Dr Sekhar Reddy, Johns Hopkins University, Baltimore, MD) and renilla (0.5 μg) using lipofectamine 2000 (Invitrogen, Life Technologies, Grand Island, NY) according to the manufacturer's instructions. After 24 h, cells were switched to 0.5% FBS-containing medium before exposure to different agents. After exposure to LY294002 (20 μM) or DMSO as a control diluent (with samples N = 3), total cell extracts were prepared and assayed for luciferase and renilla activity (Luciferase Assay System; Promega Corp., Madison, WI) using a luminometer (Berthold Technologies, Lumat, Germany). Luciferase activity was expressed as the ratio of luciferase to renilla and then normalized with respect to the control.

### Electrophoretic mobility shift assays (EMSA)

Electrophoretic gel mobility shift assays (EMSA) were used to assess binding of AP-1 to DNA and composition of AP-1 complexes. Nuclear extracts were prepared and analyzed as described by Ramos *et al.*[15]. The amount of protein in each sample was determined using the Bio-Rad protein assay (Bio-Rad, Hercules, CA). For supershift assays, nuclear extracts were incubated with antibodies to Fra-1 (Santa Cruz, CA) for 15 min at room temperature prior to addition of labeled oligonucleotide. Gels were quantitated using a Bio-Rad phosphoimager (Bio-Rad, Hercules, CA).

### Chromatin immunoprecipitation (ChIP) assays

MM cells (5 × 10^7^) were starved in medium containing 0.5% FBS overnight and then stimulated with 10% FBS for 3 h. ChIP was performed using a commercially available kit (AVIVA Systems biology, San Diego, CA). Briefly, chromatin was crosslinked by adding formaldehyde (1%) to culture medium for 15 min and sonicated. A fraction of the soluble chromatin was saved for measurement of total chromatin input. The soluble chromatin was precleared and then was immunoprecipitated with 3 μg of Fra-1 antibodies (Santa Cruz Biotechnologies), 18 h at 4°C, and the immune complexes were absorbed with protein A/G beads. Immunoprecipitated purified DNA was analyzed by semi-quantitative PCR and densitometry was used to quantify the PCR results. The *CD44 *promoter was analyzed using primers: forward 5'-tttacagcctcagcagagc-3' and reverse 5'-ggaagttggctgcagttttt-3", which yield a 184-bp DNA product.

### Real Time Quantitative PCR

Total RNA (1 μg) was reverse-transcribed with random primers using the Promega AMV Reverse Transcriptase kit (Promega, Madison WI USA.) according to the recommendations of the manufacturer. To quantify gene expression, we amplified the cDNA by TaqMan Real Time Q-PCR using the 7700 Sequence Detector (Perkin Elmer Applied Biosystems, Foster, CA). Reactions contained 1 × TaqMan Universal PCR Master Mix, 900 nM of forward and reverse primers and 200 nM of TaqMan-probes. Thermal cycling was performed using 40 cycles of 95°C for 15 s and 60°C for 1 min. Original input RNA amounts were calculated with relative standard curves for both the mRNAs of interest and the hypoxanthine phosphoribosyl transferase (*HPRT*) control. Duplicate assays were performed with RNA samples isolated from at least 2 independent experiments. The values obtained from cDNAs and *HPRT *controls provided relative gene expression levels for the gene loci investigated. The primers and probe sequences used are presented in Table 2.

**Table 2:** Primers and probes (FAM/TAMRA labeled) for Real time Q-PCR assays.

**Oligo Name**	**Primers and Probes**
FRA1-F	CTGTGCTTGAACCTGAGGCA
FRA1-R	GGTGAAAGGAGTTAGGGAGGGT
FRA1-P	TGCACACCCCCACACTCATGACC
HPRT-F	AAGCTTGCTGGTGAAAAGG
HPRT-R	AAACATGATTCAAATCCCTGA
HPRT-P	TGTTGGATTTGAAATTCCAGACAAGTTTGTT

### Transfection techniques and constructs

RK7-Fra-1Δzip, a dominant negative Fra-1 (dnFr*a*-1) construct with deletion of the leucine zipper responsible for the dimerization function of Fra-1, was obtained from Dr. M. Busslinger (Research Institute of Molecular Pathology, Vienna, Austria) and cloned into pcDNA3 (InVitrogen, San Diego, CA) before transfection using electroporation. Briefly, cells were grown to 80–90% confluence, trypsinized, counted, and resuspended at 3 × 10^6 ^cells/ml at room temperature. An aliquot of the cell suspension (400 μl) was mixed with 10 μg of plasmid DNA (expression or control plasmids) and electroporated at 280 V and 850 μF capacitance. Cells were immediately plated in fresh growth medium in 35 mm culture dishes and allowed to recover overnight. Following an overnight recovery, cells were selected for neomycin resistance using 200 μg/ml G418 (Sigma). Colonies surviving G418 selection were expanded and tested for the presence of Fra-1 as an indicator of plasmid activity. The *Fra*-1 and *CD44 *RNA interference (RNAi) duplexes were constructed from sequence information about mature mRNA extracted from the NIH genetic sequence database (GenBank^®^). The open frame region from the cDNA sequence of exon 2 of the *Fra-1 *gene, and exon 5 (common exon to all splice forms) of the *CD44 *gene. The siRNA sequences targeting *Fra-1 *corresponded to the 230–250 coding region relative to the first nucleotide of the start codon, and the sequence targeting *CD44*, corresponded to the 480–500 coding region relative to the first nucleotide of the start codon. The sequences were BLAST-searched (NCBI database) against EST libraries to ensure the specificity of the siRNA molecule. The designed oligonucleotides were inserted and structured as follows: BamHI-sense-loop-antisense-HindIII small hairpin RNA (shRNA) in the expression vector pSilencer 3.1 H1-neo siRNA (Ambion). The vector was transfected using Lipofectamine 2000 (Invitrogen) as recommended by the manufacturer. Cells were immediately plated in fresh growth medium in 35 mm culture dishes and allowed to recover overnight. Following an overnight recovery, cells were selected for neomycin resistance using 200 μg/ml G418 (Sigma St. Louis, MO). Colonies surviving G418 selection were expanded and tested for the presence of mRNA levels of *Fra-1 *and *CD44 *using 27 cycles of PCR at an annealing temperature of 57°C (*Fra-*1 forward primer: agtcaggagctgcagtgga and reverse primer: ctgctgctactcttgcgatg; *CD44 *forward primer: aagacatctaccccagcaac and reverse primer: ccaagatgatcagccattctgg and *GAPDH *control forward primer: cgggaagcttgtgatcaatgg and reverse primer: ggcagtgatggcatggactg).

### Cell motility assay

Twelve-well plastic plates were coated with 1–2 mm isotonically prepared type I/II collagen (Vitrogen 100. Cohesion, PaloAlto, CA) as recommended by the manufacturer, incubated at 37°C for 1 h to promote gelation, and dried in a laminar flow hood overnight. The dry film was then rinsed with sterile water to remove salts and re-hydrate the film. Cells were seeded on the collagen-coated plates with complete medium as described above, and grown until confluent. Cells were maintained in serumless medium for 48 h before each assay. Control experiments were performed where cell replication was inhibited 2 h prior to treatment with 1 μg/ml aphidicolin to account for cell movement due to cell growth. After treatment for 1 h with the different inhibitors, a wound was made on the coverslips using a 100 μl plastic tip, and a small 2-mm^2 ^area was marked with a template for further observations using phase contrast microscopy (Olympus M081, Olympus Industrial America Inc.) After complete medium was added, the migration of cells then was examined at 8, 24, and 48 h. A final count of cells that moved into the marked area that exhibited a spreading morphology (thin, long axis) was performed at 24 h, and the relative cell motility was estimated as a % of the area covered by cells over the total area.

### Hyaluronic acid uptake assay

Receptor-mediated internalization of BODIPY FL dye-labeled hyaluronic acid derivative was assayed as described previously [60]. Briefly, cells were grown in glass coverslips, and then treated with BODIPY FL HA conjugate probe (Molecular Probes, Carlsbad, CA, now Invitrogen) at 100 μg/ml for 2 h. Unbound probe was removed by washing 3 × with phosphate buffered saline (PBS). Cells were fixed with 3% paraformaldehyde for 10 min at room temperature and washed again with PBS. Then the slides were nuclear counterstained with DAPI (5 μg/ml solution) (Molecular Probes, Carlsbad, CA). Coverslips were mounted onto slides with AquaPolyMount (Polysciences Inc., Warrington, PA). The sections were viewed with a BioRad MRC 1024 Confocal Scanning Laser Microscope (BioRad, Hercules, CA), and images were captured in sequential mode using Lasersharp 2000 software.

### Immunohistochemistry

Human MM tissue arrays consisting of 2 mm representative areas of resected MM (N = 34) and normal lung tissue (N = 2) were obtained from Dr. Pass (New York University, NY, NY). Arrays and other samples containing formalin-fixed, paraffin-embedded samples were deparaffinized in xylene (2 × 15 min) and rehydrated in a graded ethanol series (95% to 50%). Slides were rinsed in water and placed in 1 × DAKO antigen retrieval solution (DakoCytomation, Glustrup, Denmark) and incubated at 95–99°C for 40 min. Slides then were washed 2 × for 5 min in 1 × PBS, and blocked with 50 μl of normal goat serum (Jackson ImmunoResearch Laboratories Inc., West Grove, PA) diluted in 950 μl PBS for 30 min in a humidified chamber at room temperature. A cocktail of polyclonal anti-rabbit Fra-1 antibody (R-20) (Santa Cruz Biotechnology Inc., Santa Cruz CA) diluted 1:100 and anti-mouse phosphorylated-p42/p44 (p-ERK1/2) (Cell Signaling Technology, Beverly, MA) diluted 1:100 in 1% BSA in PBS was applied to each slide and incubated overnight in a humidified chamber at 4°C. Sections then were washed in PBS and incubated for 30 min at room temperature in the dark with a secondary antibody cocktail [AlexaFluor goat anti-rabbit 568 (red), and AlexaFluor goat anti-mouse 647 (far red = blue) (Molecular Probes, Eugene, OR)]. Following a final wash in PBS, sections were counter-stained with SYTOX green (1:1,000 in PBS) or DAPI (5 μg/ml) (Molecular Probes, Eugene, OR), to detect nuclei, washed 1× in PBS, and mounted on glass slides using AquaPoly/Mount (Polysciences Inc., Warrington, PA). A negative control tissue omitting incubation with the primary antibody was included in each run.

A second set of arrays were deparaffinized as described above and double-stained with antibodies to SV40 T-antigen (Pab 101)(Santa Cruz Biotechnology Inc., Santa Cruz CA) and CD44 (Zymed/Invitrogen, Carlsbad, CA) as described previously [61]. The sections were viewed with a BioRad MRC 1024 Confocal Scanning Laser Microscope (BioRad, Hercules, CA), and images were captured in sequential mode using Lasersharp 2000 software. In addition to qualitative observations for staining, localization and extent, slides were semi-quantitatively scored for intensity on a scale of 0 to 3 using a blind coding system (data not shown).

### Statistical analyses

In all experiments, duplicate or triplicate determinations were conducted for each group per time point. Experiments were performed in duplicate. Results were evaluated by one-way analysis of variance using the Student-Newman-Keuls procedure for adjustment of multiple pairwise comparisons between treatment groups. Differences with p values ≤ .05 were considered statistically significant.

## Abbreviations

AP-1: Transcription factor, Activator protein-1;

ERK: Extracellular signal-regulated kinase;

HA: Hyaluronic acid;

MM: Malignant mesothelioma;

SV40: Simian virus 40 negative (SV40-) and positive (SV40+);

PI3K: Phosphatidyl-inositol-3-kinase;

si*Fra*-1: small interference RNA for *Fra*-1;

shFra-1: Expression vector for the small hairpin RNA directed towards Fra-1.

## Competing interests

The author(s) declare that they have no competing interests.

## Authors' contributions

MER-N designed, and performed the research. SB contributed to the confocal microscopy work. HP provided the tissue arrays and BTM contributed to the analysis and critical reading of the document.
